# Progressive Damage Behaviour Analysis and Comparison with 2D/3D Hashin Failure Models on Carbon Fibre–Reinforced Aluminium Laminates

**DOI:** 10.3390/polym14142946

**Published:** 2022-07-20

**Authors:** Haichao Hu, Qiang Wei, Boya Liu, Yun Liu, Ning Hu, Quanjin Ma, Chuancai Wang

**Affiliations:** 1 School of Mechanical and Engineering, Tianjin Sino-German University of Applied Sciences, Tianjin 300350, China; lby_woolup@163.com (B.L.); wangchuancai@tsguas.edu.cn (C.W.); 2 School of Materials Science and Engineering, Hebei University of Technology, Tianjin 300401, China; 3 School of Mechanical Engineering, Hebei University of Technology, Tianjin 300401, China; ninghu@hebut.edu.cn; 4 Beijing Zhongjia Hongsheng Power Engineering Co., Ltd., Beijing 100176, China; liuyun1415926@163.com; 5 Structural Performance Materials Engineering (SUPERME) Focus Group, Faculty of Mechanical & Automotive Engineering Technology, Universiti Malaysia Pahang, Pekan 26600, Pahang, Malaysia; neromaquanjin@gmail.com

**Keywords:** carbon fibre–reinforced aluminium laminates, CARALL, tensile properties, flexural properties, failure mechanism, 2D/3D Hashin theories

## Abstract

It is known that carbon fibre–reinforced aluminium laminate is the third generation of fibre metal materials. This study investigates the response of carbon fibre–reinforced aluminium laminates (CARALL) under tensile loading and three-point bending tests, which evaluate the damage initiation and propagation mechanism. The 2D Hashin and 3D Hashin VUMAT models are used to analyse and compare each composite layer for finite element modelling. A bilinear cohesive contact model is modelled for the interface failure, and the Johnson cook model describes the aluminium layer. The mechanical response and failure analysis of CARALL were evaluated using load versus deflection curves, and the scanning electron microscope was adopted. The results revealed that the failure modes of CARALL were mainly observed in the aluminium layer fracture, fibre pull-out, fracture, and matrix tensile fracture under tensile and flexural loading conditions. The 2D Hashin and 3D Hashin models were similar in predicting tensile properties, flexural properties, mechanical response before peak load points, and final failure modes. It is highlighted that the 3D Hashin model can accurately reveal the failure mechanism and failure propagation mechanism of CARALL.

## 1. Introduction

With the rapid development of the aerospace and automotive industries, the demand for lightweight and high-strength materials has increased. Fibre metal laminate (FML) has many advantages, such as high fatigue resistance, impact resistance, specific strength, lightweight, robust design ability, etc. [1,2]. For example, FMLs have become essential parts to the Airbus A380 fuselage [3,4,5]. However, a further application of FMLs is limited due to their low modulus and tensile strength [6]. Carbon fibre–reinforced aluminium laminate (CARALL) is the third generation of FMLs [7]. Due to the large elastic modulus of carbon fibre, CARALL has a low crack growth rate and excellent fatigue resistance. It is noted that CARALL has a higher specific strength, modulus, and impact resistance than FMLs with the same fibre content [8]. The excellent mechanical performance of CARALL has broad application prospects in aerospace, transportation, military, navigation [9], rail transit, and other fields [10]. However, due to the large gap in physical properties between aluminium matrix and carbon fibre reinforcement constituting CARALL, interface delamination and fracture can easily occur in the thermal and mechanical compound working environments [11,12]. The potential difference between aluminium alloy and carbon fibre is that it may cause electrochemical corrosion, which restricts the popularization and application of CARALL. Therefore, it is essential to explore the preparation procedure with better mechanical properties of CARALL, which also provides the mechanical properties of CARALL and predicts its failure behaviour. Research shows that the adhesive film can effectively reduce the electrochemical corrosion of the Al plate and CFRP layer [11,13,14,15,16]. The additional epoxy adhesive film interlayer can significantly improve the shear strength of laminates [17,18].

Tensile and bending tests are typical methods to evaluate the mechanical properties of composite structures [19,20,21,22,23,24,25]. For example, Lu Yao et al. [26] investigated the tensile behaviour of CARALL under various temperatures ranging from 25 °C to 175 °C, which combined experimental, theoretical, and numerical techniques. Lu Yao indicated that the tensile strength of FMLs represents a nonlinear downward trend with increasing temperature. Moreover, Changzhi Hu et al. [27] carried out a series of three-point bending tests to study the bending behaviour of different CARALLs with fibre-reinforced plastic composite/metal alternative stacking sequence. It was indicated that a comparatively high stiffness (109.70 GPa) and strength (1673 MPa) of 3/2 (three aluminium sheets and two carbon fibre/epoxy laminates) FMLs had been achieved. Wei Zhu et al. [11] prepared three types of aluminium alloy AA6061 surface treatments (sanding, anodizing, and surface modification) which improved the metal–composite interface strengths. The results were shown that a silane coupling agent modified the shear strength of the Al/CFRP/Al laminates. The results noted that the shear strengths of the laminates modified by silane coupling agent and pretreated by phosphoric acid anodizing were similar, approximately 50 MPa, which were significantly higher than those subjected to sandpaper-sanding pretreatment (38 MPa). Andrzej Kubit et al. [28] studied the impact of thermal shocks on the mechanism of composite destruction in the three-point bending test. It was found that both the failure mode and interlaminar shear strength depend on the number of thermal shock cycles.

The failure behaviours of CARALL make it challenging to capture the hidden failure modes through the experimental method before the visible failure of CARALL. Therefore, it is essential to develop both theoretical and numerical methods to predict the mechanical response of CARALL [26,27,29,30,31,32,33,34]. Recently, many researchers have developed the progressive damage analysis method, which can effectively analyse composite damage [35,36,37]. In the past decades, more than a dozen damage and failure theories of composite materials have been developed, such as maximum stress, maximum strain, Tsai Wu [38], Chang [39], Hashin [40,41], Puck criterion [42], etc. The failure criteria generally have two sorts of expressions, which includes the in-plane two-dimensional (2D) stress/strain state or the full three-dimensional (3D) stress/strain state [16]. For instance, Liu et al. [43] compared the ABAQUS built-in damage model (2D) with continuum shell elements and the 3D Hashin-type initiation criteria progressive model, which predicts the mechanical properties of countersunk bolted joint. Seo et al. [44] compared 2D and 3D failure criteria in the commercial finite element code ABAQUS for modelling stiffness degradation in the glass fibre–reinforced composite laminates. However, few studies have compared different modelling strategies of CARALL under bending loads [16].

This paper studies progressive damage behaviour analysis of carbon fibre–reinforced 6061 aluminium alloy composite laminate. The tensile and three-point bending properties are investigated using experimental and numerical methods. ABAQUS built-in two-dimensional (2D) Hashin model and the written user-defined subroutine (VUMAT) three-dimensional (3D) Hashin model are explored to predict the failure mechanism and progressive behaviour of CARALL.

## 2. Experimental Process

### 2.1. Specimen Preparation

CARALL consists of T700 carbon fibre prepreg, 6061 aluminium alloy and adhesive film. The type of prepreg used in this study was TU/T700-100/7202K-38, which was purchased from Tianjin Hanshuo High Tech Materials Co., Ltd.,Tianjin, China. Several parameters of this CFRP material are mainly provided in Table 1. The type of the aluminium alloy was EN AW-6061-O. “O” represents the annealing state, which means that the alloy has been completely annealed. The strength of aluminium alloy in this state is generally low. The 6061 aluminium alloy plates were provided by Guangxi Nannan aluminium processing Co., Ltd., Guangxi Province, China. The chemical composition of 6061 aluminium alloy are shown in Table 2. In order to reduce the effect of electrochemical corrosion, layers of the adhesive film are added at the interface between aluminium plate and carbon fibre prepreg. The type of adhesive film is HX-F125. The adhesive film is supplied by Tera Max Material Co., Ltd., Shanghai, China. Several parameters of this adhesive film are mainly provided in Table 3.

The thickness of the carbon fibre prepreg is 0.125 mm, and the thickness of the 6061 aluminium alloy plate is 0.5 mm. The carbon fibre prepreg and aluminium alloy plate are laid alternately, and the layer is arranged in the way of complete symmetry of [Al/0°/90°/90°/0°/Al/0°/90°/90°/0°/Al]. In this study, the surface of 6061 aluminium alloy was anodised. The specific process is as follows:

Pretreatment of aluminium alloy surface: polish the aluminium plate with 400 # sandpaper, wash with water, and degrease in 25 °C sodium hydroxide aqueous solution for 1 min. Then, use deionised water to wash quickly to avoid the defect of alkali washing flow mark. Place nitric acid aqueous solution at 25 °C for 1 min, and carefully wash the aluminium alloy plate with deionised water to remove grease, dirt, and dust.

Anodizing of aluminium alloy: the phosphoric acid solution is used as the electrolyte of the anodising process. Graphite or a stainless steel plate is used as the cathode, and aluminium alloy plate to be treated is used as anode. Put the pretreated aluminium alloy plate in step (1) in the configured phosphoric acid aqueous solution at 25 °C for anodic oxidation. In the oxidation process, the power supply voltage is 10 V, the current is controlled at 2.7 A, the anodic oxidation time is 10–15 min, and a certain thickness of the coating is produced. When the anodizing process is completed, the coated aluminium alloy plate is washed with deionised water and dried in air.

**Figure 1:**
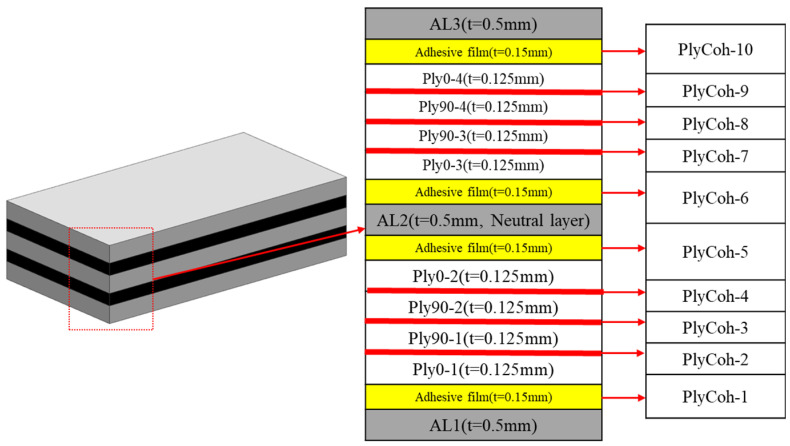
Schematic diagram of CARALL specimens.

**Figure 2:**
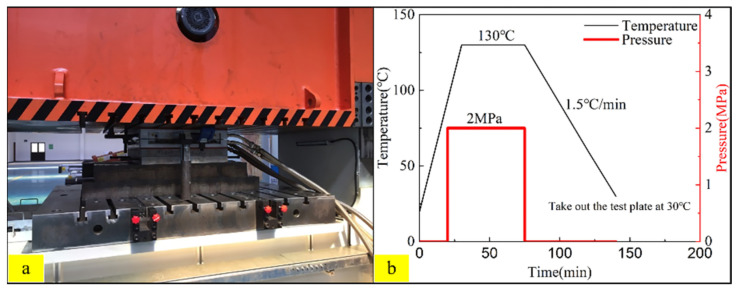
Moulding equipment and curing process parameters (**a**) Moulding equipment (**b**) Curing procedure with temperature and pressure parameters.

A schematic diagram of the CARALL structure is shown in Figure 1. The moulding process is used prepared CARALL, and the curing procedure is illustrated in Figure 2.

### 2.2. Tensile and Three-Point Bending Tests

**Figure 3:**
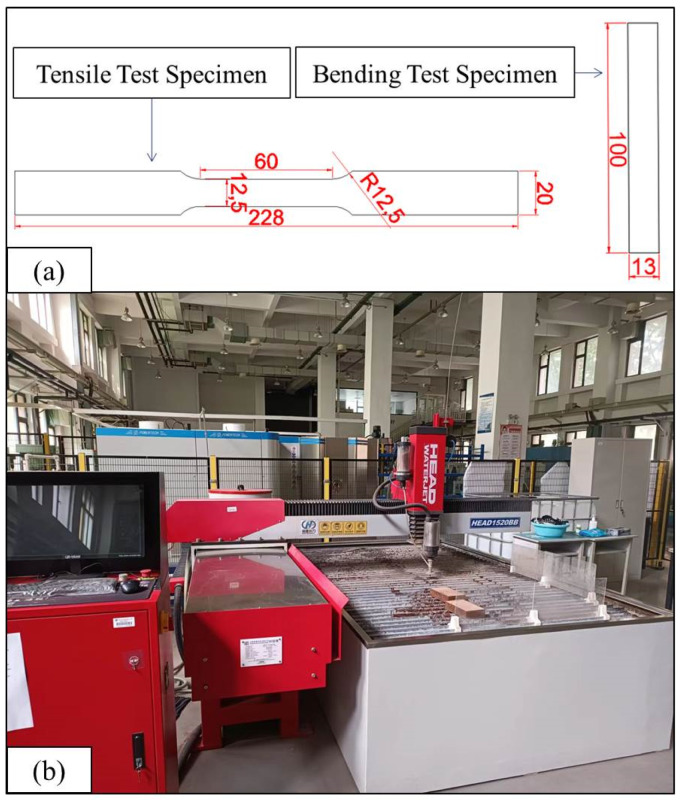
Sample size diagram and water jet cutting equipment (**a**) Schematic dimension of tensile and three-point bending test specimens (**b**) Water jet cutting equipment.

**Figure 4:**
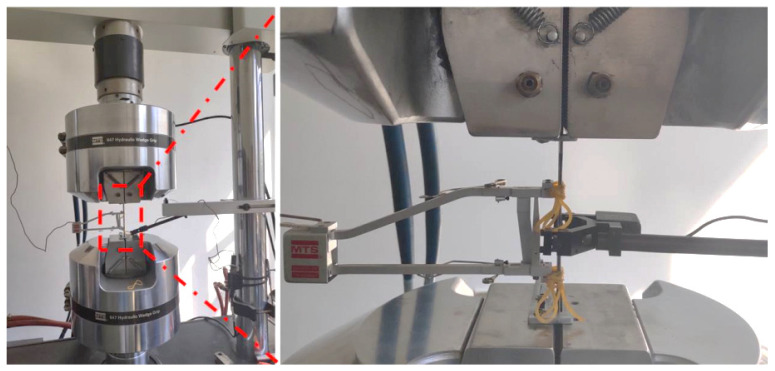
Experimental setup of the tensile test [35].

Based on the ASTM-D638 standard test method and ASTM-D790 standard test method, the specimens of tensile test and bending test were prepared and cut through the water jet cutting equipment. The water jet cutting equipment and the parameters of the specimens are shown in Figure 3. Water jet cutting is a standard method to process fibre metal composite laminates. The cutting speed of water jet cutting is about 1200 mm/min. The water flow pressure is 420 Mpa. The sand volume is 0.5 kg/min. The tensile test is performed through MTS electronic universal testing machine, as shown in Figure 4.

**Figure 5:**
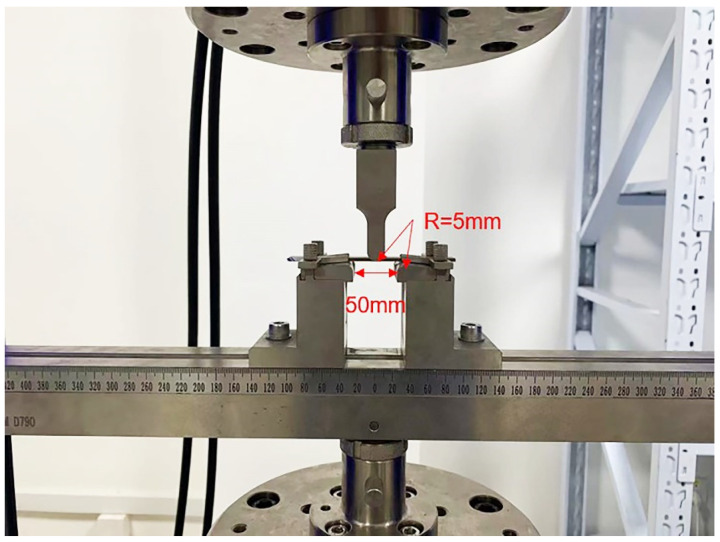
Experimental setup of the three-point bending test.

The three-point bending test is referred to as the ASTM-D790 standard test method. Then, the prepared composite laminate is cut into 100 × 13 × 3.1 mm through water jet cutting. Furthermore, the bending test is performed using MTS electronic universal testing machine. Figure 5 illustrates the experimental setup of the three-point bending test. The span dimension is 50 mm, and the radius of the two supporters is 5 mm.

Bending properties are studied based on several parameters, such as bending stress, bending strain, and bending modulus, which are calculated as in the following three formulas:

(1) $$\sigma_{f}=\frac{3PS\;}{2bh^{2}}$$

(2) $$\;\varepsilon_{f}=\frac{6Dh\;}{S^{2}}$$

(3) $$E_{f}=\frac{S^{3}\;m}{4bh^{3}}$$

where *P* is the load at a given point at the load-defection curve and *b* is the width of the specimen. Moreover, *D* is the deflection at the middle point of the specimen and *m* is the slope of the tangent to the initial straight-line portion of the load-deflection curve.

### 2.3. Characterization Observation of Fracture Morphology

Characterization observation of the specimens was carried out after the tensile and three-point bending tests. The fracture morphology of the tensile test was observed through the ZEISS SUPRATM 55 scanning electron microscope.

## 3. Progressive Damage Models of CFRP/Al Composite Laminates

### 3.1. Johnson–Cook Model for 6061 Aluminium Alloy Layers

The modelling of the aluminium layer used the Johnston–Cook model to predict the mechanical response of the aluminium layer without considering the influence of temperature. The constitutive model is:

(4) $$\sigma =\left[A+{B\varepsilon }_{p}^{n}\right]\left[1+\mathrm{Cln}\frac{\dot{\varepsilon }}{{\dot{\varepsilon }}_{0}}\right]$$

where A, B, and C are material parameters, $\varepsilon_{p}$ is the equivalent plastic strain, n is the material constant, and $\frac{\dot{\varepsilon }}{{\dot{\varepsilon }}_{0}}$ is the dimensionless plastic strain rate. In order to model the ductile failure of the aluminium layer, the damage parameter φ is introduced:

(5) $$\varphi =\sum \left(\frac{\Delta {\bar{\varepsilon }}_{\mathrm{pl}}}{{\bar{\varepsilon }}_{f}^{\mathrm{pl}}}\right)$$

$\Delta {\bar{\varepsilon }}_{\mathrm{pl}}$ is the equivalent plastic strain increment, ${\bar{\varepsilon }}_{f}^{\mathrm{pl}}$ is the equivalent plastic strain at the beginning of damage, and its expression is:

(6) $${\bar{\varepsilon }}_{f}^{\mathrm{pl}}=\left(d_{1}+d_{2}e^{-d_{3}\eta }\right)\left(1+d_{4}\ln\frac{{\dot{\bar{\varepsilon }}}_{\mathrm{pl}}}{{\dot{\varepsilon }}_{0}}\right)$$

where η is the stress triaxiality parameter, $d_{1}\sim d_{4}$ are material parameters, as summarised in Table 4.

### 3.2. Failure Criteria for CFRP Layer

Hashin developed the three-dimensional (3D) Hashin failure criterion based on the two-dimensional (2D) failure criterion in 1980. The 2D Hashin criterion has been integrated into ABAQUS. In this paper, the VUMAT subroutine is written to study the damage mechanism of the laminated layers using the 3D Hashin criterion. The expression is provided as follows:

(7) $$\mathrm{FT}:F_{f}^{t}={\left(\frac{\varepsilon_{11}}{\varepsilon_{11}^{T}}\right)}^{2}+\alpha {\left(\frac{\varepsilon_{12}^{2}+\varepsilon_{13}^{2}}{\gamma_{12}^{2}}\right)}^{2}\geq 1,\varepsilon_{11}^{T}=\frac{X^{T}}{E_{1}}\left(\varepsilon_{11}\geq 0\right)$$

(8) $$\mathrm{FC}:F_{f}^{c}={\left(\frac{\varepsilon_{11}}{\varepsilon_{11}^{C}}\right)}^{2}\geq 1\left(\varepsilon_{11}\leq 0\right),\varepsilon_{11}^{C}=\frac{X^{C}}{E_{1}}\;\left(\varepsilon_{11}\leq 0\right)$$

(9) $$\mathrm{MT}:F_{m}^{t}=\frac{{\left(\varepsilon_{22}+\varepsilon_{33}\right)}^{2}}{\;{\left(\varepsilon_{22}^{T}\right)}^{2}}+\frac{\varepsilon_{12}^{2}+\varepsilon_{13}^{2}}{\gamma_{12}^{2}}+\frac{\varepsilon_{23}^{2}-\varepsilon_{22}\varepsilon_{33}}{\gamma_{23}^{2}}\geq 1\left(\varepsilon_{22}\geq 0\right)\;\left(\varepsilon_{22}\geq 0\right)$$

(10) $$\mathrm{MC}:F_{m}^{c}=\left[{\left(\frac{\varepsilon_{22}^{c}}{2\gamma_{23}}\right)}^{2}-1\right]\frac{\varepsilon_{22}+\varepsilon_{33}}{\varepsilon_{22}^{T}}+{\left(\frac{\varepsilon_{22}+\varepsilon_{33}}{2\gamma_{23}}\right)}^{2}+\frac{\varepsilon_{12}^{2}+\varepsilon_{13}^{2}}{\gamma_{12}^{2}}+\frac{\varepsilon_{23}^{2}-\varepsilon_{22}\varepsilon_{33}}{\gamma_{23}^{2}}\geq 1\;\left(\varepsilon_{22}\leq 0\right)$$

(11) $$\varepsilon_{22}^{T}=\frac{Y^{T}}{E_{2}},\varepsilon_{22}^{C}=\frac{Y^{C}}{E_{2}},\gamma_{12}=\frac{S_{12}}{G_{12}},\gamma_{23}=\frac{S_{23}}{G_{23}}$$

where $X^{T}$, $X^{C},{\;Y}^{T},{\;Y}^{C}$ are the tensile strength in the fibre direction, compressive strength in the fibre direction, tensile strength in the matrix direction, and compressive strength in the matrix direction of the composite monolayer, respectively. $S_{12},{\;S}_{13},{\;S}_{12}$ are the shear strength in different planes of laminated layers. The T modelling parameters of carbon fibre composites are shown in Table 5.

### 3.3. 2D and 3D Hashin Progressive Damage Model Description

The ABAQUS-embedded 2D Hashin criterion can describe the damage of composite materials in in-plane stress states. However, it cannot predict the progressive damage in three-dimensional stress states. In practice, the damage of composite materials is complex, which predicts that the failure behaviour of composite materials in three-dimensional stress state is close to the actual damage situation.

In order to comprehensively compare the similarities and differences between ABAQUS-embedded 2D Hashin criterion and 3D Hashin criterion in practical application, the VUMAT composite damage subroutine is written in FORTRAN language, and the 3D Hashin criterion is written in the process. Therefore, the stiffness degradation of the composite after entering the damage state needs to be considered. The damage variable d is used as the sign of the damage state. When the damage variable d is 0, the material does not enter the damaged state. The material fails when the damage variable d = 1. When d is between 0 and 1, the composite is in the stiffness degradation stage. The composite material has entered the damage state but has not entirely failed yet. The composite stiffness matrix is transformed into the stiffness degradation matrix related to the damage variable by introducing the damage variable. These stress parameters are calculated according to the damage variable M, which is provided as follows:

(12) $$\hat{\sigma }=M\cdot \sigma$$

(13) $$M=\left[\begin{array}{ccc} 1/\;(1-d_{f}) & 0 & 0\\ 0 & 1/\;(1-d_{m}) & 0\\ 0 & 0 & 1/\;(1-d_{s}) \end{array}\right]$$

where $d_{f}$, ${\;d}_{m}$, $d_{s}$ are the damage variables related to fibre, matrix and shear damage, respectively, and which are derived from the failure modes of CFRP:

(14) $${\;d}_{f\;}=\left\{\begin{array}{c} d_{f}^{t}\;\mathrm{if}\;{\;\hat{\sigma }}_{11}\geq 0\\ d_{f}^{c}{\;\mathrm{if}\;\hat{\sigma }}_{11}<0 \end{array}\right.$$

(15) $$d_{m}=\left\{\begin{array}{c} d_{m}^{t}{\;\mathrm{if}\;\hat{\sigma }}_{22}\geq 0\\ d_{m}^{c}{\;\mathrm{if}\;\hat{\sigma }}_{22}<0 \end{array}\right.$$

(16) $$d_{s}=\;1-(1-d_{f}^{t})\;(1-d_{f}^{c})\;(1-d_{m}^{t})\;(1-d_{m}^{c})$$

When the damage initiation criterion is reached, further loading leads to the degradation of material stiffness. As a function of the damage variable, the stiffness matrix of damage is summarised as follows:

(17) $$C_{d}=\frac{1}{\Omega }\left[\begin{array}{ccc} \left(1-d_{f}\right)E_{1} & \left(1-d_{f}\right)\left(1-d_{m}\right)\nu_{12}E_{1} & 0\\ \left(1-d_{f}\right)\left(1-d_{m}\right)\nu_{12}E_{1} & \left(1-d_{f}\right)E_{2} & 0\\ 0 & 0 & \left(1-d_{S}\right)G_{12}d_{f} \end{array}\right]$$

(18) $$\Omega =\left(1-d_{f}\right)\;\left(1-d_{m}\right)\nu_{12}\nu_{21}$$

**Figure 6:**
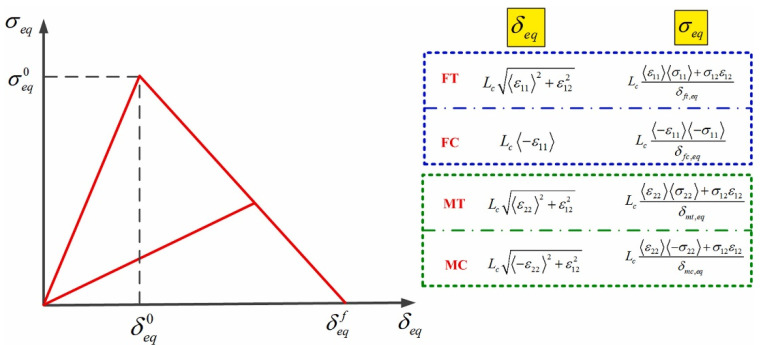
The constitutive relationship of composite materials in the 2D Hashin model [16].

In order to reduce the dependence on the mesh during material stiffness degradation, the ABAQUS built-in 2D Hashin model introduces a characteristic length equivalent to the square root of the shell element $L_{c}$. The constitutive relationship under the four modes can be expressed as the equivalent stress displacement relationship. Figure 6 illustrates the typical linear evolution law curve, the expression of equivalent displacement, and equivalent stress. After the damage starts, the damage variable under a specific mode is obtained by the following formula:

(19) $$d=\frac{\delta_{i,\mathrm{eq}}^{f}\;(\delta_{i,\mathrm{eq}}-\delta_{i,\mathrm{eq}}^{0})}{\delta_{i,\mathrm{eq}}\;(\delta_{i,\mathrm{eq}}^{f}-\delta_{i,\mathrm{eq}}^{0})}{\;\delta }_{i,\mathrm{eq}}^{0}\leq \delta_{i,\mathrm{eq}}\leq \delta_{i,\mathrm{eq}}^{f};\;i∋\;\left(\mathrm{fc},\mathrm{ft},\mathrm{mc},\mathrm{mt}\right)$$

$\delta_{i,\mathrm{eq}}^{0}$ the equivalent damage initial failure displacement under specific failure mode. $\delta_{i,\mathrm{eq}}^{f}$ is the equivalent displacement when the material is completely damaged in a specific mode. It can be determined by fracture energy $G_{i,c}$ and equivalent stress $\sigma_{i,\mathrm{eq}}^{0}$:

(20) $$\delta_{i,\mathrm{eq}}^{f}=\frac{G_{i,c}}{\sigma_{i,\mathrm{eq}}^{0}}$$

**Figure 7:**
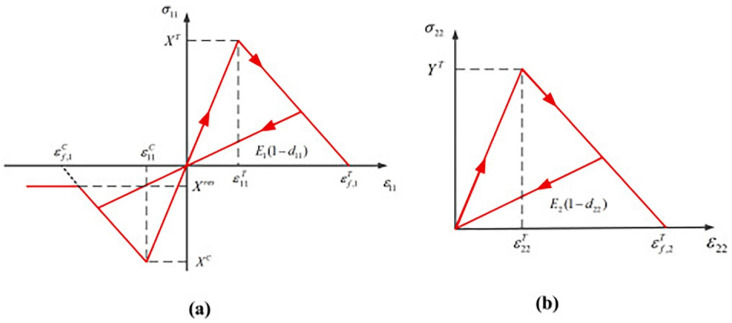
The linear relationship between fibre tensile and compression damage in the 3D Hashin VUMAT subroutine [16].

The damage subroutine of the 3D Hashin VUMAT model adopts the bilinear evolution law. The evolution diagram of fibre tensile and compression damage is shown in Figure 7. The evolution model of matrix tensile and compression damage is the same as the fibre model, but the calculation model of the damage variable is different. The specific formula is provided as follows:

(21) $$\left\{\begin{array}{c} d_{\mathrm{ft}\;\left(c\right)}=\frac{\varepsilon^{T\;\left(C\right)}{}_{f,1}}{\varepsilon^{T\;\left(C\right)}{}_{f,1}-\varepsilon_{11}^{T\;\left(C\right)}},\varepsilon^{T\;\left(C\right)}{}_{f,1}=\frac{2G_{\mathrm{ft}\;\left(c\right)}}{X^{T}L_{c}}\;\;\;\;\\ d_{\mathrm{mt}\;\left(c\right)}=\frac{\varepsilon^{T\left(C\right)}{}_{m,2}\;\left(\varepsilon_{22}-\varepsilon_{22}^{T\;\left(C\right)}\right)}{\varepsilon_{22}\;(\varepsilon^{T\;\left(C\right)}{}_{m,2}-\varepsilon_{22}^{T\left(C\right)})},\varepsilon^{T\left(C\right)}{}_{m,2}=\frac{2G_{\mathrm{mt}\;\left(c\right)}}{Y^{T}L_{c}} \end{array}\right.$$

**Figure 8:**
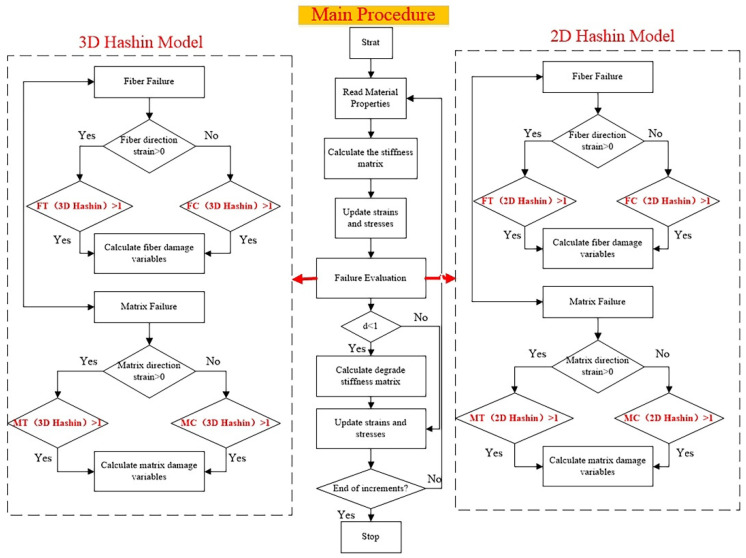
Flow chart of simulation procedure between 2D Hashin and 3D Hashin VUMAT models.

$\varepsilon^{T\;\left(C\right)}{}_{f,1}$ and $\varepsilon^{T\left(C\right)}{}_{m,2}\;$ are the critical failure strain under a specific mechanism. The flow chart of the simulation procedure between 2D Hashin and 3D Hashin VUMAT models is compared and summarised in Figure 8.

### 3.4. Failure Criteria and Damage Evolution Law for Interface

The cohesive element model is widely used to model delamination in composite laminates. Based on the traction separation criterion, the constitutive relationship of cohesive force element idealises the complex fracture mechanics problem. The specific expression is as follows:

(22) $$\left(\begin{array}{c} t_{n}\\ t_{s}\\ t_{t} \end{array}\right)=\left[\begin{array}{ccc} K_{nn} & 0 & 0\\ 0 & K_{ss} & 0\\ 0 & 0 & K_{tt} \end{array}\right]\left(\begin{array}{c} \delta_{n}\\ \delta_{s}\\ \delta_{t} \end{array}\right)$$

where $t_{n}$, $t_{s}$, $t_{t}$ are the nominal traction stress in the thickness direction, first, and second shear direction. $K_{nn}$, $K_{ss}$, $K_{tt}$ and $\delta_{n}$, $\delta_{s}$, $\delta_{t}$ are the penalty stiffness and equivalent displacement corresponding to the above directions.

(23) $${\left\{\frac{\left\langle t_{n}\right\rangle }{t_{n}^{0}}\right\}}^{2}+{\left\{\frac{t_{s}}{t_{s}^{0}}\right\}}^{2}+{\left\{\frac{t_{t}}{t_{t}^{0}}\right\}}^{2}=1$$

Here, $t_{n}^{0}$*,* $t_{s}^{0}$*,* $t_{t}^{0}$ represents the Mode I, Mode II, and Mode III failure strength. After the interfacial failure initiates, the stress components of the traction-separation model are degraded the damage variable $D_{c}$ in the form of:

(24) $$t_{n}=\left\{\begin{array}{c} \left(1-D_{c}\right)\bar{t_{n}}\left(\bar{t_{n}}\geq 0\right)\\ \bar{t_{n}}\;\; \end{array}\right.$$

(25) $$\;t_{s}=\left(1-D_{c}\right)\bar{t_{s}}$$

(26) $$t_{t}=\left(1-D_{c}\right)$$

The mixed-mode linear energy-based damage propagation criterion is used, and it is the Benzeggagh–Kenane (BK) law. The fracture energy $G_{c}$ is given by:

(27) $$G_{c}=G_{IC}+\left(G_{IIc}-G_{c}\right){\left(\frac{G_{shear}}{G_{Ic}+G_{shear}}\right)}^{\eta }$$

(28) $$G_{shear}=G_{IC}+G_{IIIC}$$

where *G_IC_*, *G_IIC_*_,_ and *G_IIIC_* are Mode I, Mode II, and Mode III fracture toughness, respectively, and η is the mixed-mode parameter.

The effective displacement $\delta_{m}$ is introduced to account for the damages caused by the combined effects of normal and shear deformations.

(29) $$\delta_{m}=\sqrt{\langle \delta_{m}{}^{2}\rangle +\delta_{s}^{2}+\delta_{t}^{2}}$$

Therefore the damage variable $D_{c}$ during a linear degradation stage can be calculated as:

(30) $$D_{c}=\frac{\delta_{m}^{f}\left(\delta_{m}^{max}-\delta_{m}^{0}\right)}{\delta_{m}^{max}\left(\delta_{m}^{f}-\delta_{m}^{0}\right)}$$

(31) $$\delta_{m}^{f}=2G_{c}/T_{\mathrm{eff}}$$

where $\delta_{m}^{f}$ is the effective displacement at complete failure with $T_{\mathrm{eff}}\;$ as the adequate traction at damage initiation. $\delta_{m}^{max}$ refers to the maximum effective displacement attained during the analysis. Material properties of the cohesive layers adopted from the published literature are listed in Table 6.

### 3.5. Discretization of CARALL Specimens

**Figure 9:**
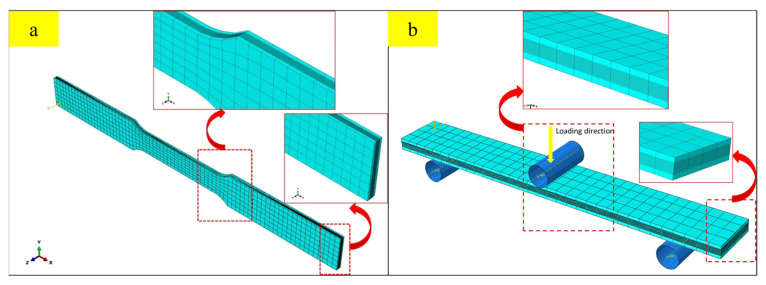
Finite element modelling used in this study: (**a**) modelling of the tensile specimen; (**b**) modelling of the three-point bending specimen.

For composite layers, eight-node continuum shell elements (SC8R) were used in ABAQUS built-in 2D Hashin model, and eight-node solid brick elements (C3D8R) were used in the 3D Hashin VUMAT model. Aluminium layers were also modelled with elements C3D8R. The cohesive bonding layer part adopts the 8-node three-dimensional bonding element COH3D8. It is considered the computational time and accuracy, and the general mesh size was decided to be 2 × 2 mm. The area under the loading nose and the chamfer position of the tensile specimen was refined to be 0.5 × 0.5 mm. The detailed element model of the tensile and three-point bending tests was presented in Figure 9. The thickness and stacking sequence of aluminium-T6 alloy plates, carbon fibre layer and adhesive film are set according to Figure 1. The adhesive film (PlyCoh-1,5,6,10) between the aluminium alloy layer and the fibre layer is modelled by cohesive elements. The unit thickness is set to 0.1 mm. The material performance parameters are mainly determined according to Table 3 and Table 6. The interface between the fibre layers (PlyCoh-2,3,4,7,8,9) is simulated by the cohesive elements, the thickness of which is set to 0.01 mm.

## 4. Results and Discussion

### 4.1. Experimental Analysis of Tensile Test

**Figure 10:**
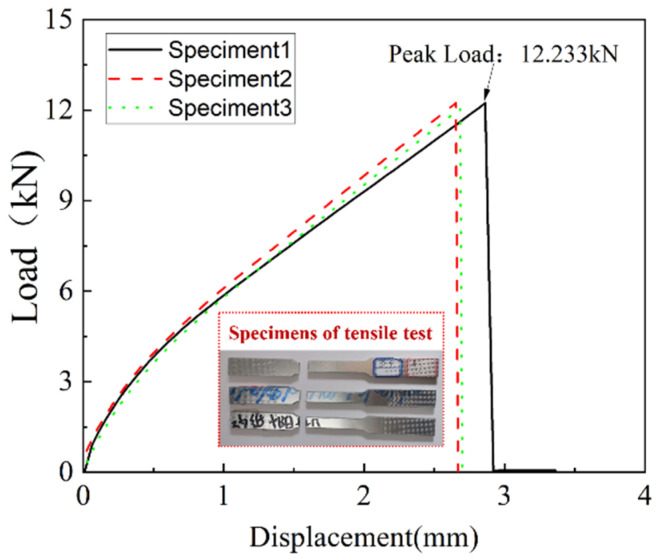
Load versus displacement curves of tensile test.

Three tensile specimens were conducted in this study. The load versus displacement curves of the tensile test are shown in Figure 10. Before fracture failure, the load versus displacement curve showed the apparent bilinear behaviour. When it reached the ultimate load, the load suddenly decreased to zero, and the experiment was terminated. The three experimental results show that the ultimate load of CARALL under uniaxial tension was around 12 kN. The peak load is 12.23 kN. The ultimate displacement is close to 3 mm. The specimens were observed in the brittle fracture mode, and there was no obvious shrinkage deformation at the fracture.

**Figure 11:**
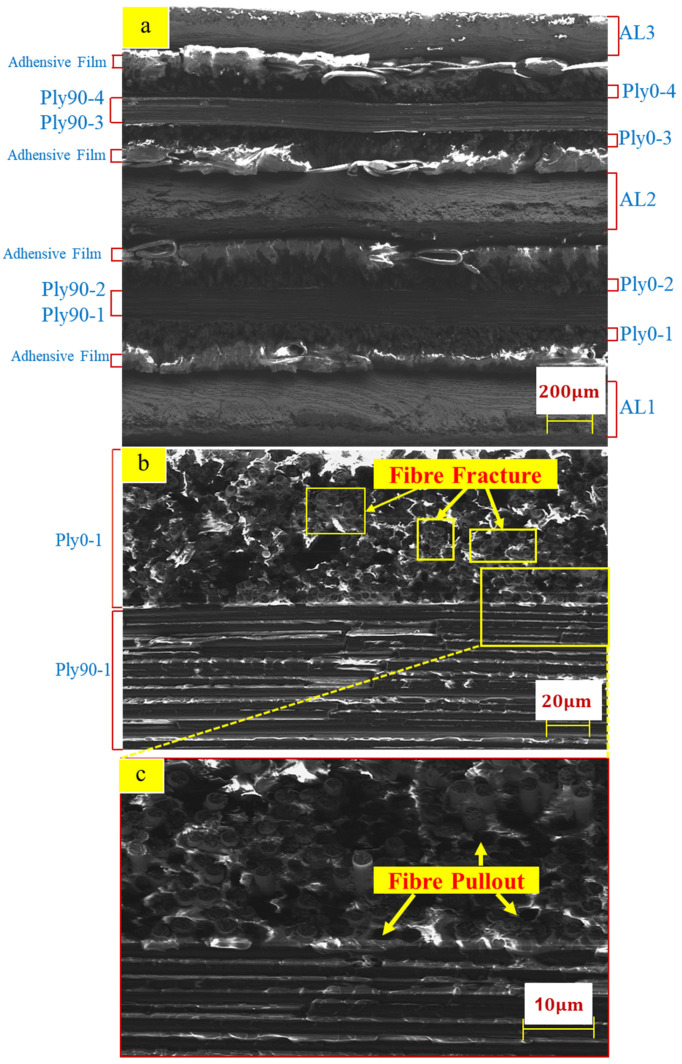
SEM morphologies of tensile specimen fracture (**a**) overall cross-section (**b**) SEM morphologies of the Ply0-1 and Ply90-1 layers (**c**) Partially enlarged view of Ply0-1 and Ply90-1.

Figure 11 shows the SEM morphologies of tensile specimens. Furthermore, Figure 11a presents the overall microstructure of the fracture, which is relatively flat. The adhesive film inserted between the aluminium alloy and carbon fibre interface can be observed. Figure 11b,c illustrates the carbon fibre and matrix presented brittle fracture and failure. Parts of the fibre bundles in Ply0-1 were pulled out, and parts of the fibre bundles in Ply0-1 were stretched to fracture.

In conclusion, the evident damage modes were captured from the SEM observation: fibre pulls out, fibre fracture, and matrix fracture.

### 4.2. Comparison between Tensile Test Results, 2D, and 3D Hashin Models

**Figure 12:**
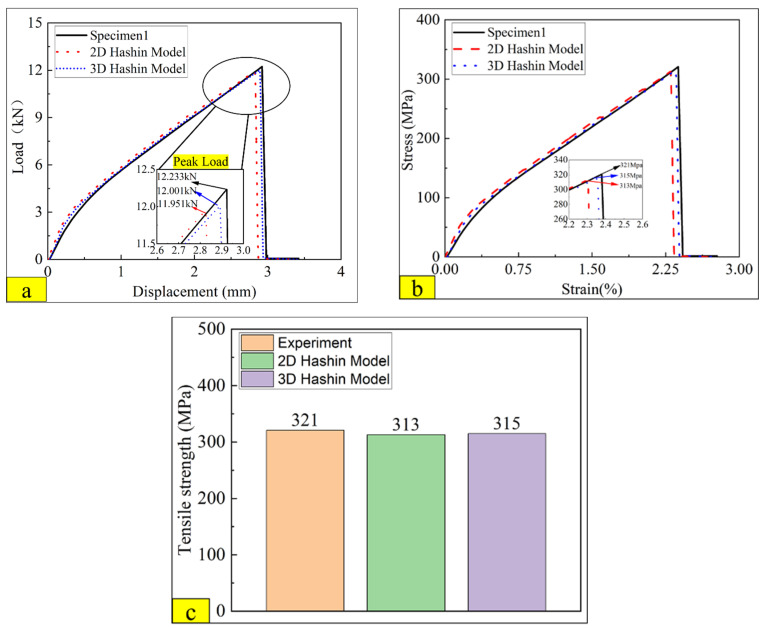
Comparison results of the tensile test on 2D and 3D Hashin model (**a**) load versus displacement curves comparison (**b**) true strain-stress curves comparison (**c**) tensile strength comparison.

Figure 12a compares experimental load versus displacement curve on 2D and 3D Hashin numerical results. It can be seen that both the 2D Hashin model and the 3D VUMAT model can predict the apparent bilinear behaviour of CARALL before reaching the ultimate load, which is closed to the experimental results. The fracture point of CARALL is predicted accurately on both the 2D and 3D Hashin models. The peak load of the specimen on the 2D Hashin model and 3D Hashin model are 12.23 kN,12.00 kN, and 11.95 kN, respectively. The load versus displacement curve decreases suddenly after reaching the peak load. The predicted peak load of the 2D Hashin model and 3D Hashin model is close to the experimental result. Figure 12b compares the true strain versus true stress from the experimental and the numerical results on the 2D Hashin model and 3D Hashin model. Figure 12c compares the tensile strength of the experimental and the numerical results on the 2D Hashin model and 3D Hashin model. The tensile strength values are 321 MPa, 313 MPa, and 315 MPa, respectively. The predicted results of both numerical models are all very close to the experimental results.

**Figure 13:**
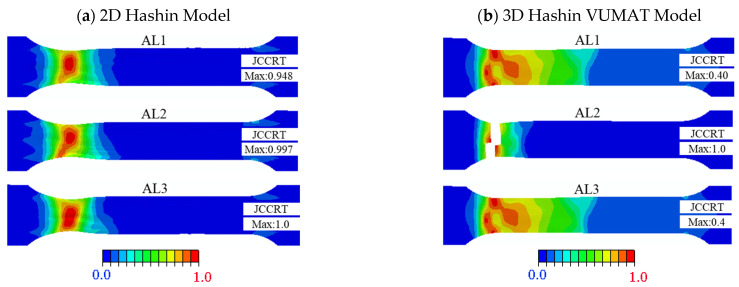
Failure cloud diagram of the aluminium alloy specimen: (**a**) 2D Hashin model; (**b**) 3D Hashin VUMAT model.

Figure 13 presents the failure patterns of aluminium alloy completed on the 2D Hashin model and 3D Hashin model. The two models are predicted that the aluminium near the fillet is close to complete failure, and the rest is not in the damaged state. The prediction results are consistent with the fracture positions as the experimental results. For the 3D Hashin VUMAT model, there is an apparent fracture position of the AL2 aluminium alloy plate, which is exactly close to the fracture position of the aluminium alloy in the experimental tests.

**Figure 14:**
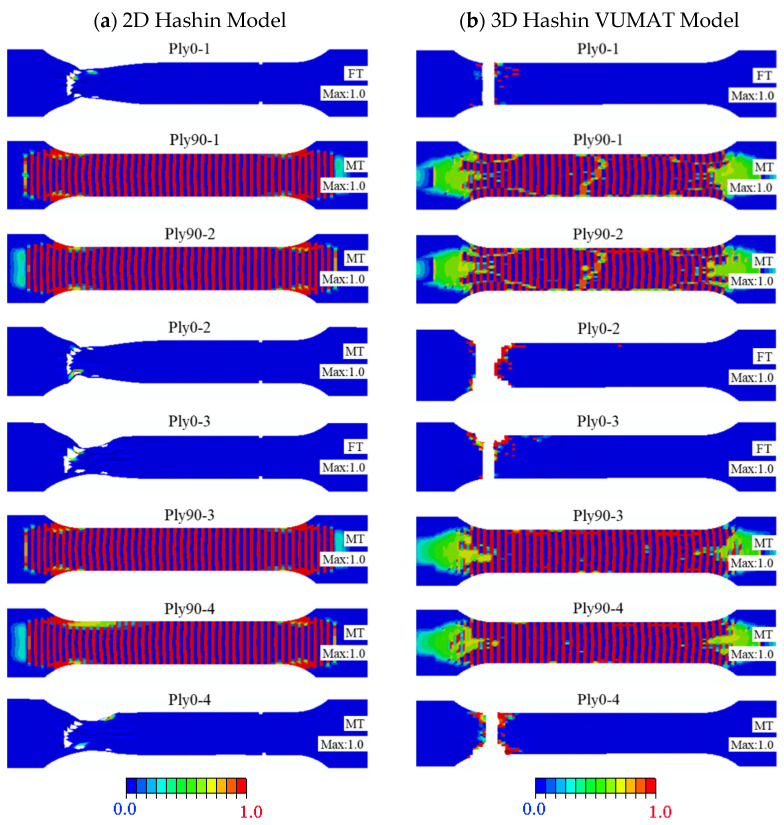
The final damage patterns of carbon fibre layers: (**a**) 2D Hashin model (**b**) 3D Hashin model.

Figure 14 illustrates the damage and failure patterns comparison results of the carbon fibre layer completed by the 2D Hashin model and 3D Hashin model. Both models can present large-area damage to the carbon fibre layer. For example, Ply0-1, Ply0-2, Ply0-3, and Ply0-4 show apparent fibre tensile fracture failure. The fracture locations predicted by the two models are reasonably close to the experimental results. Damage to the fibre in the 0° direction was discovered next to the fracture section, while other positions did not enter the damage state. These are consistent with the fracture morphologies from the experimental test, as shown in Figure 11.

Ply90-1, Ply90-2, Ply90-3, and Ply90-4 of the two models have large-area matrix tensile failure, which is observed from the experimental specimens. However, there is no apparent fracture section found in the experiment results. This phenomenon may be because the stress concentration mainly causes the fracture location in the experimental works. The fracture of the finite element modelling is controlled through the equation, which is generally recognised as fracture behaviour if it exceeds the given value.

Unlike the 2D Hashin model, the 3D Hashin model can predict that 0° layer fibre does not have shrinkage deformation in the fracture area, and the model size is almost unchanged until the fracture failure. This phenomenon occurs due to the 2D Hashin model, which determines the grid deletion by the damage variable. Once the damage variable reaches “1”, the grid is deleted, regardless of the failure caused by excessive grid distortion. For the 3D Hashin model, the maximum principal strain and the minimum principal strain are introduced as additional element deletion criteria to prevent grid distortion’s influence on the convergence of the model. The introduction of this criterion makes the results predicted by the 3D model, which is more accurate than the 2D Hashin model.

**Figure 15:**
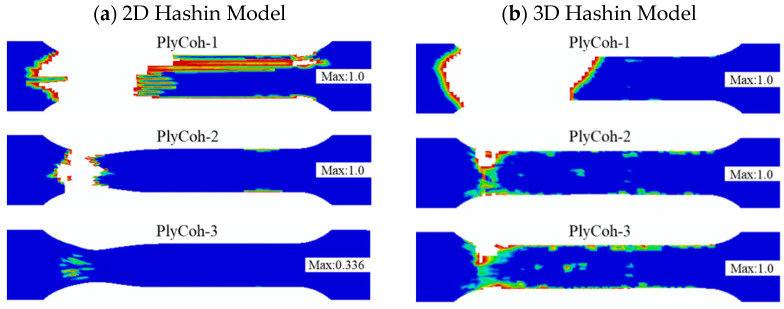

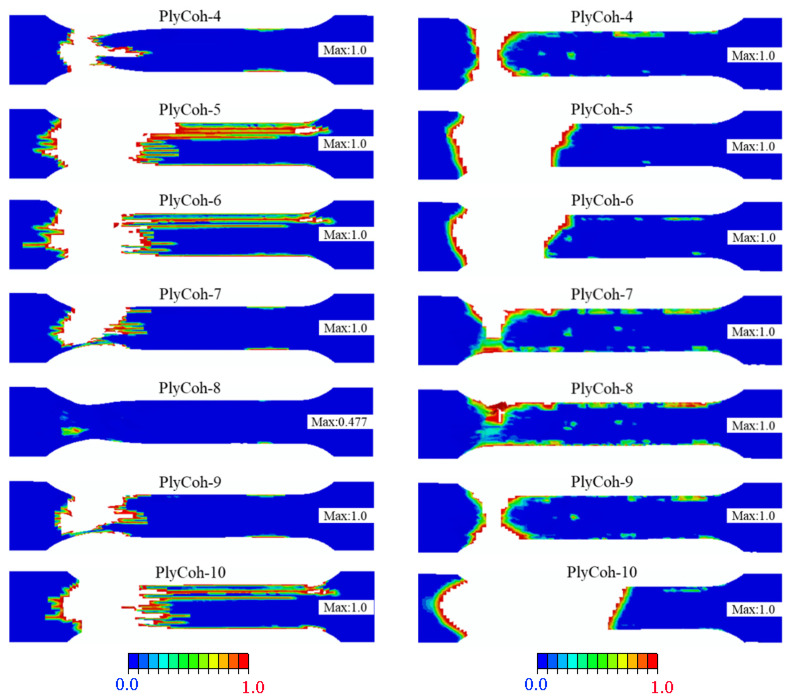
The final damage patterns of cohesive layers: (**a**) 2D Hashin model; (**b**) 3D Hashin model.

Figure 15 presents the failure patterns of the interlaminar damage failure calculated by the 2D Hashin model and 3D Hashin model. The prediction results of the two models are roughly the same results. The interlaminar of aluminium alloy layer and 0° carbon fibre (Plycoh-1, Plycoh-5, Plycoh-6, Plycoh-10) presents interlaminar delamination failures. The fibre fracture location can detect apparent delamination failure between 0° and 90° carbon fibre layers (Plycoh-2, Plycoh-4, Plycoh-7, Plycoh-9). In general, the prediction results of the two models are reasonably in good agreement with the experiment results.

### 4.3. Experimental Analysis of the Three-Point Bending Test

**Figure 16:**
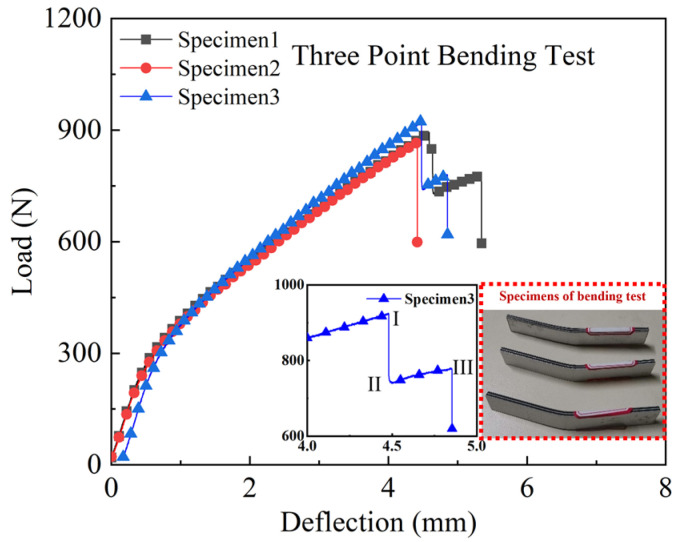
Load versus deflection curves of the three-point bending tests on the CARALL.

Three specimens were carried out for the three-point bending test. The experimental load-deflection curves are shown in Figure 16. Before reaching the ultimate load, the load-deflection curves have apparent bilinear behaviour. The bending failure deflection interval of CARALL in three specimens is 4 to 5 mm. The maximum bending load is 925 N, and there are two evident decline stages in the load-deflection curve, which are labelled as three fracture points I, II, and III in Figure 16. After the second sudden drop of loading, the experiment is terminated. The phenomenon of decline stages should be related to the layer-by-layer failure of carbon fibre in the bending process.

**Figure 17:**
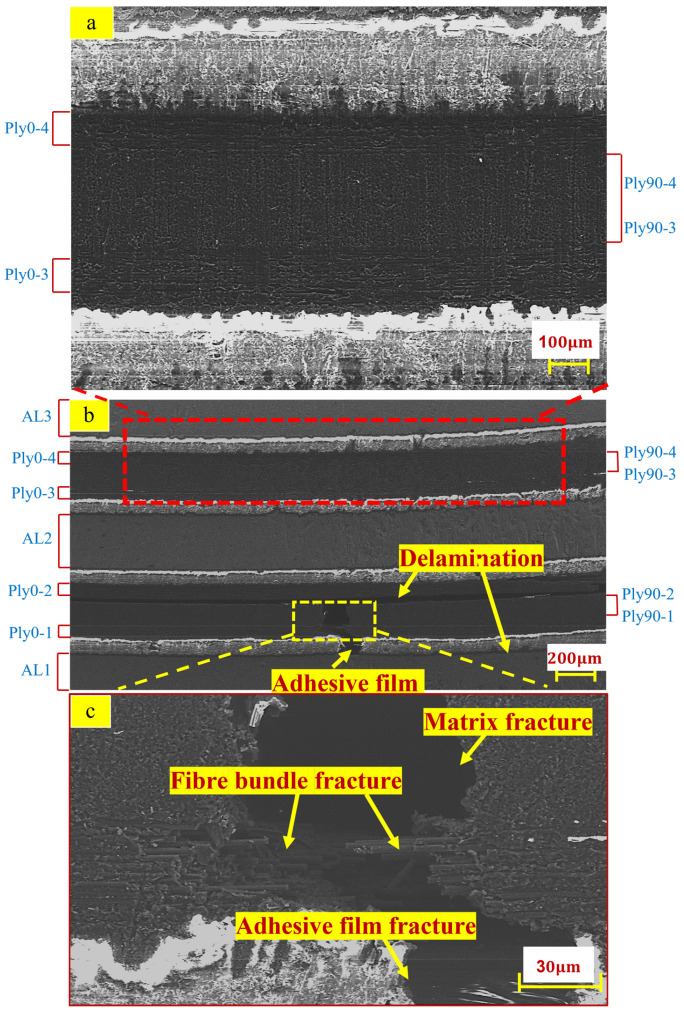
Failure morphology of the three-point bending specimen: (**a**) partially enlarged view above neutral layer; (**b**) overall cross-sectional view; (**c**) partially enlarged view of the failure location below the neutral layer.

Figure 17a illustrates a partially enlarged view above the neutral layer. There is no apparent damage between the 0° layer fibre and the 90° layer matrix above the neutral layer. Figure 17b illustrates the damage position of the laminate under the three-point bending load, which mainly occurs in the carbon fibre part below the neutral layer. There are mainly three failure modes: delamination of carbon fibre interfacial failure, cracking of 90° layer’s matrix, fracture of 0° layer’s fibre bundle, and rupture of adhesive film between aluminium alloy and carbon fibre. There is a large-area delamination failure found in cohesive layers PlyCoh-1 and PlyCoh-4. Figure 17c demonstrates the partially enlarged view of the failure location below the neutral layer. The matrix of Ply90-1 and Ply90-2 layers are shown brittle fracture failure. The fibre bundle of the Ply0-1 layer is broken and released, which results in the fracture failure adhesive film (PlyCoh-1).

### 4.4. Comparison between Three-Point Test Results, 2D, and 3D Hashin Models

**Figure 18:**
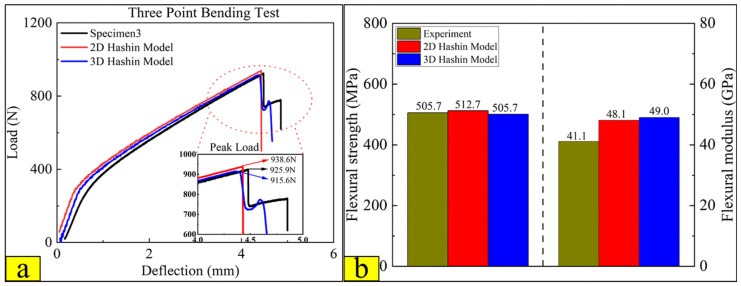
Comparison results between experimental, 2D, and 3D Hashin on three-point bending test: (**a**) load-deflection curves; (**b**) flexural strength and flexural modulus.

Figure 18 compares the experimental test, 2D, and 3D Hashin on the flexural response. Then, the curves predicted by the 2D and 3D Hashin models are obviously bilinear, which is consistent with the experimental results. Figure 16 labels the three fracture points (I, II, and III) after reaching the ultimate load. The 2D and 3D Hashin models have accurately captured the peak point (Point I). The peak bending load of the experimental test, 2D and 3D Hashin models are 925.9 N, 938.6 N, and 915.6 N, respectively.

The load-deflection curve predicted by the 2D Hashin model decreases suddenly after reaching the peak load. Moreover, the 3D Hashin model shows a decline after fracture failure, which has good agreement with the experimental results. It is obtained that the 3D Hashin model is more accurate for capturing breakpoints II and III. For example, Yuan Lin [16] noted that the neglect of the contribution made by the out-of-plane stress components causes the incapability of the 2D Hashin model, which reveals the accurate failure propagation features. Figure 18b compares the flexural strength and modulus of the experimental test, 2D Hashin, and 3D Hashin models, respectively. The flexural strength and modulus of the experimental tests are 505.7 MPa and 41.1 Gpa, respectively. The predicted results of the two models are close to the experimental results.

According to the experimental test, the damage failure mainly occurs in the carbon fibre and interface layers below the neutral layer. Therefore, the failure behaviour of CARALL after entering the damage state is further analysed through the damage cloud diagram corresponding to the three fracture points on the 3D Hashin model load-displacement curve.

**Figure 19:**
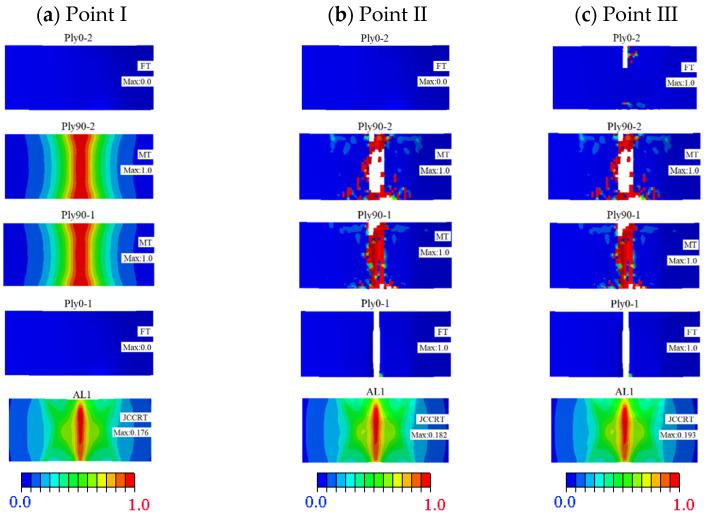
Damage program of the carbon fibre layer and the aluminium layer below the neutral layer at different fracture points: (**a**) Point I; (**b**) Point II; (**c**) Point III.

Figure 19a illustrates the damage cloud diagram of each layer below the neutral layer at fracture point I. The carbon fibre layers of Ply90-1 and Ply90-2 took the lead in the relatively concentrated matrix tensile fracture damage directly below the loading indenter. It can be seen that the matrix tensile fracture failure of Ply90-1 and Ply90-2 has become the main factor for the occurrence at Point I. Figure 19b shows the damage cloud diagram at fracture point II. Compared with Figure 19a–c illustrates that when Ply0-1 appears to have the tensile fracture failure and Point II is observed. However, Ply0-2 has not entered the damage state. Ply0-2 is the primary bearing object of the fibre layer between fracture points II and III. With increasing the bending loading, the damage of Ply0-2 intensifies, as shown in Figure 19c. While the damage of Ply0-2 begins to expand along the width direction until it extends to the whole width, the load-deflection curve suddenly decreases after fracture point III.

**Figure 20:**
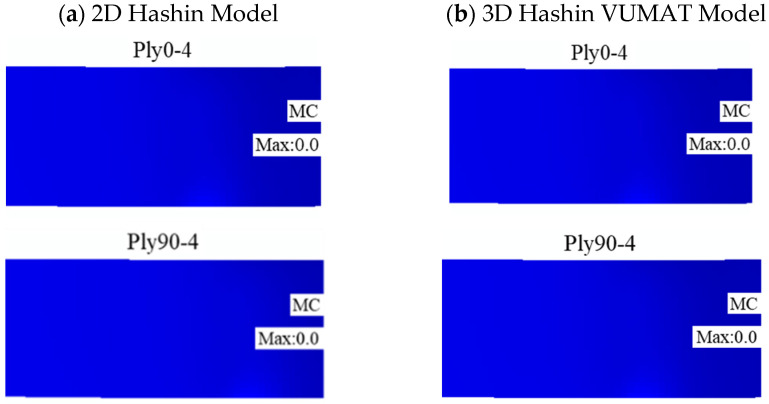

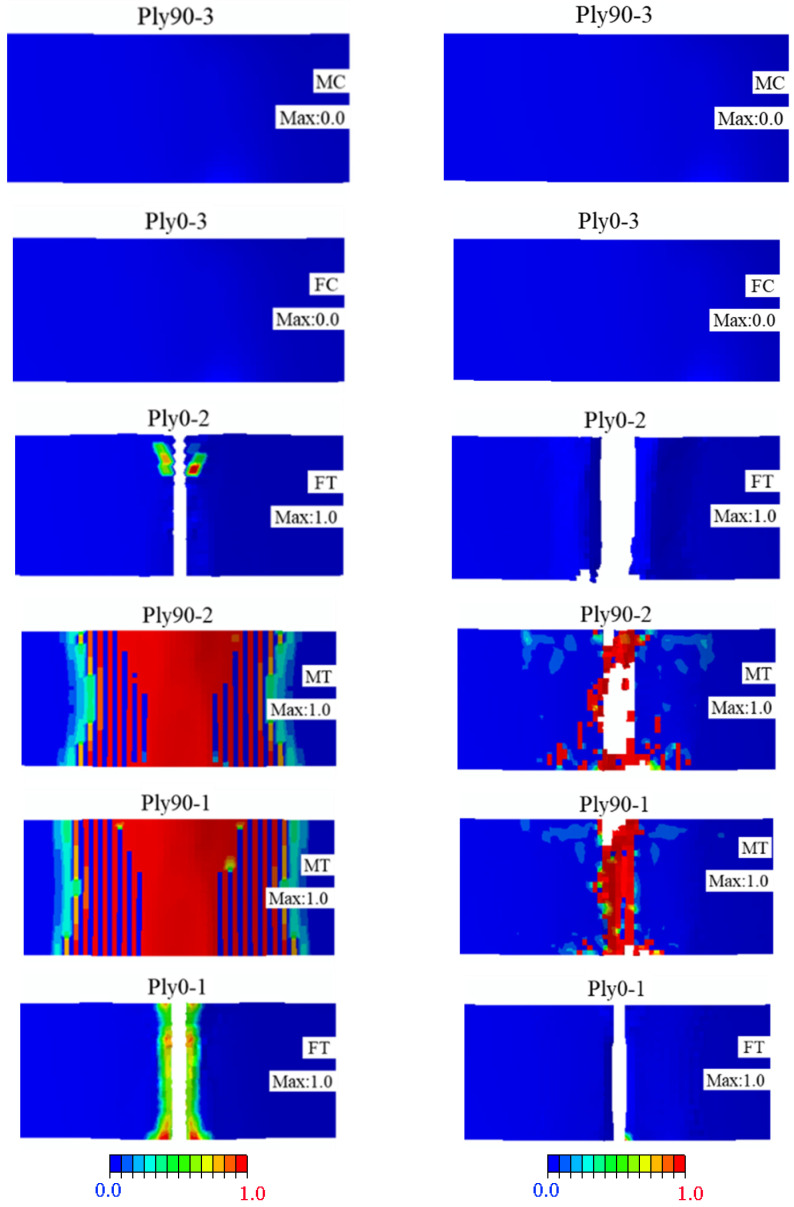
Damage patterns of the carbon fibre layer: (**a**) 2D Hashin model; (**b**) 3D Hashin model.

Figure 20 illustrates the final damage patterns of the carbon fibre layer calculated by the 2D and 3D Hashin models. The predicted results of the two models are similar, and the carbon fibre layer above the neutral layer does not enter the damage state. The predicted results have a good agreement with the results. However, there are apparent differences between the two models in predicting damage and failure results of Ply90-1 and Ply90-2. Although both models can predict the in-plane tensile failure of Ply90-1 and Ply90-2 carbon fibres, the 2D Hashin model predicts that the damage and failure pattern has a large area of matrix damage and failure damage expands around. Compared to the 2D Hashin model, the 3D Hashin model predicts that the location of the matrix failure in the damage program is relatively concentrated. The failure area is a bit smaller than the 2D Hashin model, which is more consistent with the experimental results, as shown in Figure 17. It may be because the 2D Hashin failure criterion does not consider the out-of-plane shear deformation and its interaction with matrix damage.

**Figure 21:**
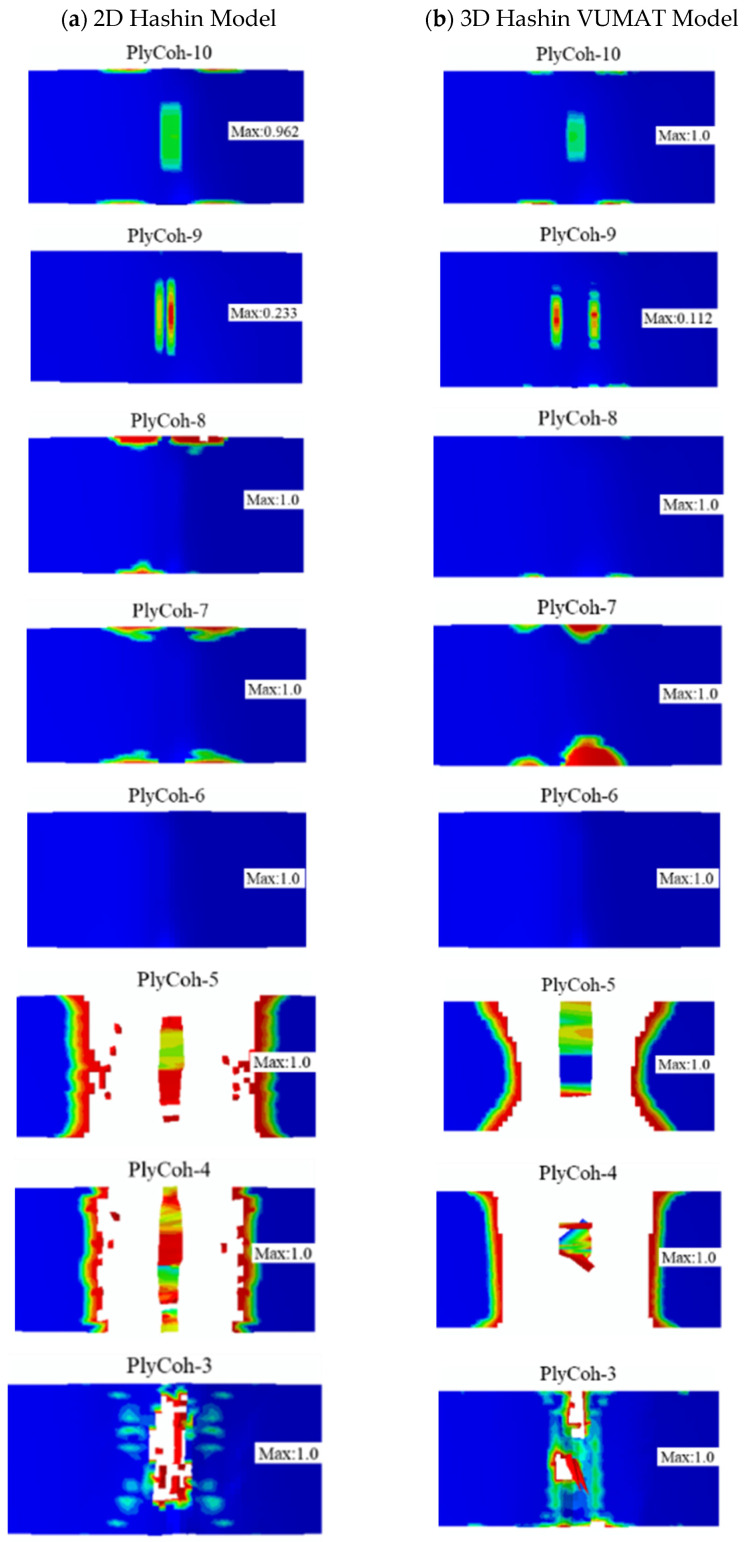

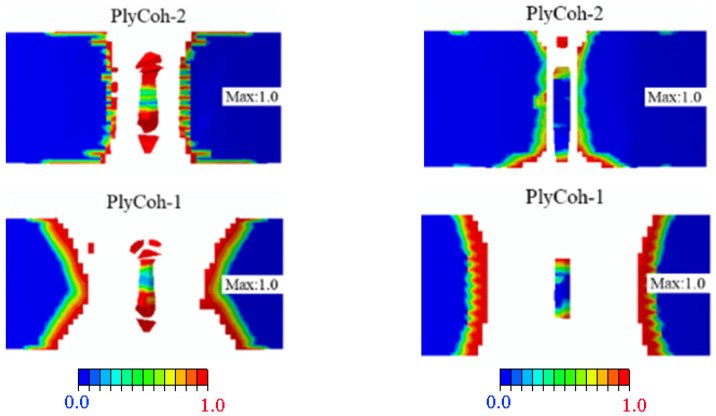
The final interlayer damage patterns: (**a**) 2D Hashin model; (**b**) 3D Hashin model.

Figure 21 shows the final failure patterns of the final damage interface between CARALL layers predicted by the 2D and 3D Hashin models. Above the neutral layer, there is no apparent delamination failure at the interface of CARALL. Due to the local transverse compression effect, interlayer interfaces Plycoh-10 and Plycoh-9 observe partial interlaminar damage. The delamination damage failure occurs below the neutral layer, and the failure positions are mainly located directly below the indenter, in which Plycoh-5, Plycoh-4, and Plycoh-1 are particularly damaged. The failure areas of Plycoh-3 and Plycoh-2 are smaller than Plycoh-5, Plycoh-4, and Plycoh-1. The failure area of Plycoh-2 predicted by the 3D Hashin model is more concentrated than that of the 2D Hashin model. It may be due to the overestimation of the fibre damage area of Ply90-1 and Ply90-2 layers by the 2D Hashin model. The results predicted by the 3D Hashin model are much closer to the micromorphology of the fracture observed in the experimental results.

## 5. Conclusions

This paper investigates the tensile and three-point bending properties of CARALL using experimental and numerical methods. For finite element modelling analysis, the 2D Hashin and 3D Hashin VUMAT models are used to compare and analyse the composite layers. A bilinear cohesive contact model simulates the interface failure, and the Johnson–Cook model describes the aluminium layer. The mechanical response and failure analysis of CARALL were evaluated through load versus deflection curves. The numerical results were studied and analysed. Due to the limitations of this study, the main conclusions are summarised as follows:

(1) Microscopic observation shows that the failure forms of CARALL under the tensile loading mainly show aluminium layer fracture, fibre pull-out and fracture, and matrix tensile fracture. The load versus displacement curve of tension is obviously bilinear. It is noted that the load decreases rapidly after reaching the peak load. The peak load is 12.23 kN, and the tensile strength is 321 MPa.
(2) The failure response of CARALL under three-point bending loading mainly exists below the neutral axis, and the failure modes are as follows: matrix fracture failure (Ply90-1 and Ply90-2), fibre fracture (Ply0-1), adhesive film failure (PlyCoh-1) and delamination failure (PlyCoh-1, PlyCoh-4). The load-displacement curve presents bilinearity before reaching the ultimate strength, and after reaching the ultimate strength, the damage evolution stage has three obvious breaking points in the stepped decline. The peak flexural loading, flexural strength, and modulus are 925.9 N, 505.7 MPa, and 41.1 GPa, respectively.
(3) The 2D Hashin and 3D Hashin models provide similar capabilities in predicting typical tensile and flexural properties before peak load points and final failure modes. Out-of-plane stress components are considered in the 3D Hashin model, and additional element deletion is introduced to calculate the damaged area, which is more accurate and avoids large deformation of the mesh. It is highlighted that the 3D Hashin model successfully predicted the step drop phenomenon in the load-displacement curve in the bending test. It is revealed that the step drop is mainly due to the failure of Ply90-1, 2, Ply0-1, and Ply0-2. Therefore, the 3D Hashin model revealed the failure mechanism and failure propagation of the CARALL more accurately.

## Figures and Tables

**Table 1:** Several parameters of T700 carbon fibre prepreg.

Properties	Value
Curing temperature (°C)	120~140
Glass transition temperature (Tg/°C)	95
Weave pattern	Unidirectional pattern (UD)
Fibre surface density (g/m^2^)	100 ± 5
Resin content (%)	38 ± 3
Recommended forming process	Vacuum bag moulding process Compression Moulding

**Table 2:** Chemical composition of 6061 aluminium alloy (wt%).

Si	Fe	Cu	Mn	Mg	Cr	Zn	Ti	Al
0.68	0.5	0.33	0.12	0.9	0.28	0.05	0.02	97.12

**Table 3:** Several parameters of the adhesive film.

Properties	Value
Curing temperature (°C)	130–150
Film thickness (mm)	Before curing: 0.24 ± 0.02 After curing: 0.1 ± 0.01
The surface density of adhesive film (g/m^2^)	300 ± 20
Shear strength at 25 °C (MPa)	40

**Table 4:** 6061 aluminium alloy used Johnson–Cook model parameters [16,45].

Parameters	Material Parameters
Elastic parameters	E =70,000 Mpa, μ=0.3
Tensile strength	205 MPa
Yield surface parameter	A=252MPa, B =426 MPa, C=0.015, m=1, η=0.34 n =0.34
Failure parameters	$d_{1}$$=0.13,\;d_{2}$$=0.13,\;d_{3}$$=1.5,\;{\;d}_{4}$= 0.011
Fracture energy	${\;G}_{\mathrm{Ic}}=8\;k$ $J/m^{2}$

**Table 5:** Carbon fibre composite material parameters [46].

Parameters	Material Parameters
Elastic modulus	$E_{1}=120,000\;\mathrm{MPa},\;{\;E}_{2}=E_{3}=7800\;\mathrm{MPa}$
Poisson’s ratio	$\nu_{12}=\nu_{13}=\nu_{23}=0.3$
Shear modulus	$G_{12}=G_{13}=4000\;\mathrm{Mpa}$ $,\;G_{23}=3600\;\mathrm{MPa}$
Density	$\rho =2000\;\mathrm{kg}/m^{3}$
Ultimate strength	$X^{T}=1800\;\mathrm{MPa},\;\;X^{C}=1250\;\mathrm{Mpa}$ $,\;Y^{T}=Z^{T}=50\;\mathrm{MPa},$ $Y^{C}=Z^{C}=150\;\mathrm{Mpa}$ $,\;S_{12}=S_{13}=93\;\mathrm{MPa},{\;S}_{23}=50\;\mathrm{MPa}$
Fracture energy	$G_{11}^{T}=G_{11}^{C}=40\;\mathrm{kJ}/m^{2}$ $,\;G_{22}^{T}=0.25\;\mathrm{kJ}/m^{2}$ $,\;G_{22}^{C}=0.75\;\mathrm{kJ}/m^{2}$

**Table 6:** Material properties of cohesive layers [16].

Properties	Value
$\rho \;\left(\mathrm{Kg}/m^{3}\right)$	1200
$K_{\mathrm{nn}}^{0}{=K}_{\mathrm{ss}}^{0}{=K}_{\mathrm{tt}}^{0}\left(N/{\mathrm{mm}}^{3}\right)$	10^6^
$t_{n}^{0}\left(\mathrm{MPa}\right)$	40
$t_{s}^{0}{=t}_{t}^{0}\left(\mathrm{MPa}\right)$	50
$G_{\mathrm{IC}}\left(N/\mathrm{mm}\right)$	0.25
$G_{\mathrm{IIC}}\left(N/\mathrm{mm}\right)$	0.75
$G_{\mathrm{IIIC}}\left(N/\mathrm{mm}\right)$	0.75
η	1.45

## Data Availability

All data presented in this paper are available upon reguest from the corresponding author.
